# Effect of plant bio-stimulants on productivity, bio-energy efficiency and profitability of a berseem-pearl millet cropping system

**DOI:** 10.3389/fmicb.2026.1776509

**Published:** 2026-03-26

**Authors:** Marthala Bhuvaneswar Reddy, Devendra Kumar Dadhich, Harjeet Singh, Dibyajyoti Pramanik, Vrushabh Vijay Fiskey, Sukeswara Achari Kamsali, Tejaswini Chandrakar, Shruti Setia, Sudha Yadav, Mahendra Vikram Singh Rajawat, Kamal Garg, Sanjeev Kumar

**Affiliations:** 1 ICAR-Indian Veterinary Research Institute, Bareilly, Uttar Pradesh, India; 2 ICAR-National Dairy Research Institute, Karnal, Haryana, India; 3 Department of Agronomy, Iowa State University, Ames, IA, United States; 4 Crop Bioengineering Center, Iowa State University, Ames, IA, United States; 5 Dhanuka Agritech Research and Technology Center, Dhanuka Agritech Limited, Gurgaon, Haryana, India

**Keywords:** energy auditing, fodder, food, integrated nutrient management, PGPR, seaweed extract, system productivity, VAM

## Abstract

A field study was conducted during the Rabi-Summer seasons of 2023-24 and 2024-25 at Fodder Farm Section, ICAR-IVRI, to assess the impact of bio-stimulants based crop nutrient management on berseem-pearl millet (fodder-food) cropping system on alluvial soils of IGP of western India. The treatments tests were framed with inclusion of three major plant bio-stimulants [plant growth-promoting rhizobacteria (PGPR), vesicular arbuscular mycorrhiza (VAM), and seaweed extract (SWE)] with four levels of recommended dose of fertilizers (RDF) (0, 50, 75 and 100%) as T_1_—Absolute control, T_2_—PGPR + VAM, T_3_—PGPR + VAM + 50% RDF, T_4_—PGPR + VAM + 75% RDF, T_5_—PGPR + VAM + 3 sprays of SWE, T_6_—PGPR + VAM + 50% RDF + 2 sprays of SWE, T_7_—PGPR + VAM + 75% RDF + 1 sprays of SWE, T_8_—100% RDF, T_9_—PGPR + VAM + 100% RDF and T_10_—PGPR + VAM + 100% RDF + 1 sprays of SWE. The treatments were tested adopting RBD with five replications. The experimental findings revealed that the inclusion of plant bio-stimulants in conventional nutrient management, i.e., treatment T_9_ and T_10_, significantly enhanced the system productivity in terms of green fodder yields, dry fodder yields and system equivalent yields. Also, the same treatments registered significantly improved bio-energy efficiency in terms of energy output, energy productivity and profitability. Economic feasibility assessment further revealed that T_9_ and T_10_ are statistically at par with respect to system gross returns, net returns and B:C ratio. The results solidify the agronomic and economic argument for using bio-stimulants to meet future fodder and food needs, energy security, and sustainability of intensive fodder-based food systems in India.

## 1 Introduction

Ensuring food and fodder security for India’s rapidly growing human and livestock populations while conserving natural resources is a major challenge of the 21st century. The human population is increasing at an annual rate of 1.60%, while livestock population growth stands at 0.66%, intensifying competition for limited agricultural land. Projections indicate that by 2050, India will require nearly 400 million tonnes of food annually, necessitating a sustained agricultural growth rate of at least 4%. Simultaneously, the livestock sector faces severe feed deficits, estimated at 35.6% for green fodder, 10.95% for dry fodder, and 44% for concentrates (Koli and Bhardwaj, 2018). Despite rising demand, only about 5.3% of the cultivated area is allocated to fodder crops, a figure that has remained largely unchanged for decades (Koli and Bhardwaj, 2018).

Despite the recognized importance of integrating fodder crops into intensive production systems, current agricultural practices remain heavily dependent on cereal-based monocropping supported by high external inputs, particularly chemical fertilizers (Sharma et al., 2019; Chimonyo et al., 2023). This input-intensive approach increases production costs, reduces energy-use efficiency, and contributes to environmental degradation, including soil health deterioration and nutrient imbalances (Sharma et al., 2019; Alam et al., 2024). Although legume inclusion and biological inputs such as plant bio-stimulants have shown promise in enhancing nutrient use efficiency and system sustainability (Ananda et al., 2022), their combined role in improving productivity and energy efficiency within fodder-based cropping systems has not been adequately quantified. In particular, there is a lack of system-level evidence on how graded fertilizer doses integrated with microbial and seaweed-based bio-stimulants influence bio-energy efficiency, economic returns, and input substitution potential in intensively managed fodder–food cropping sequences under the western Indo-Gangetic Plains. Addressing this knowledge gap is essential for designing low-input, energy-efficient, and economically viable cropping systems capable of meeting future food and fodder demands without exacerbating resource depletion.

Energy, nutrients, and water constitute the principal inputs governing agricultural productivity and sustainability (Saad et al., 2016; Jat et al., 2019). With increasing intensification of cropping systems, the relationship between energy input and agricultural output has gained prominence, particularly under resource-constrained environments (Chaudhary et al., 2017). Among all energy inputs, chemical fertilizers contribute the largest share (44–54%) of total input energy, followed by land preparation, fuel consumption, and human labour (Chaudhary et al., 2009; Kargwal et al., 2019). Excessive reliance on chemical fertilizers not only escalates production costs but also reduces energy efficiency and environmental sustainability.

Inclusion of leguminous fodder crops in intensive cropping systems has been shown to enhance energy use efficiency by reducing nitrogen fertilizer requirements while improving overall system productivity and profitability (Mohanty et al., 2024). Furthermore, integration of plant bio-stimulants such as PGPR, VAM, and seaweed extracts with inorganic fertilizers has demonstrated potential to improve nutrient acquisition, reduce external input dependence, and enhance energy efficiency in cropping systems (Ali et al., 2021; Chatzistathis et al., 2024; Mondal et al., 2025). However, information remains limited on the combined impact of bio-stimulants and graded fertilizer doses on productivity, bio-energy efficiency, and economic performance of fodder-based cropping systems in the western Indo-Gangetic Plains.

The PGPR and VAM have been extensively recognized as effective biological control agents, capable of suppressing soil-borne pathogens, inducing systemic resistance, and mitigating disease severity in crops (Basu et al., 2021; Sun et al., 2024; Al Raish et al., 2025). PGPR consortia have been shown to reduce disease incidence in tomato and other horticultural crops, while VAM associations enhance plant immunity and modulate rhizosphere microbial communities, limiting pathogen proliferation (Lahlali et al., 2022; Oulad Ziane et al., 2024). In addition, seaweed extracts contain bioactive compounds that act as elicitors of plant defense responses, complementing the protective effects of microbial inoculants and contributing to sustainable disease management (Raja and Vidya, 2023; Suji et al., 2024; Sekar et al., 2025). Collectively, the integration of these biological agents offers an eco-friendly and effective alternative to chemical pesticides, promoting crop health, resilience, and sustainability in intensive agricultural systems. This study presents a novel, system-level evaluation of bio-stimulant-based nutrient management by simultaneously assessing productivity, energy efficiency, and profitability in a berseem–pearl millet cropping system. It is among the first to demonstrate that microbial and seaweed-based bio-stimulants can partially substitute chemical fertilizers without compromising yield or economic returns, while improving energy-use efficiency and sustainability under intensive fodder systems.

It was hypothesized that diversification of cereal-based cropping systems with a legume fodder–cereal food system (berseem–pearl millet), coupled with the integration of plant bio-stimulants and inorganic fertilizers, would reduce energy input requirements while enhancing system productivity, bio-energy efficiency, and profitability in a sustainable manner.

## 2 Materials and methods

### 2.1 Experimental site, climate and soil

The field experiment was conducted during the Rabi-summer seasons of 2023-24 and 2024-25 at the ICAR-Indian Veterinary Research Institute fodder farm in Izatnagar, Uttar Pradesh. The site lies on the Northern Gangetic alluvial plains at 28°24′N, 79°25′E, and an altitude of 176 m. The climate features a bimodal annual rainfall of 950–1,005 mm, with over 70% occurring from July to September. Relative humidity ranges from 20% in the dry summer months to 96–98% during the monsoon. Maximum temperatures peak at 45 °C in May-June, while minimums drop to 5 °C in December-January. Soil samples were taken from a depth of about 0 to 15 cm by following all guidelines prescribed for taking soil samples (Peck and Melsted, 1967) and analysed at the Agronomy Laboratory, ICAR-NDRI, Karnal by adopting the standard protocol. The experimental field soil had a sandy loam texture with a neutral to slightly alkaline pH of 7.2, and moderately fertile, being medium in soil organic carbon (0.55%), low in available nitrogen (194.92 kg·ha^−1^), medium in available potassium (207.56 kg·ha^−1^) and slightly high in soil available phosphorus (28.09 kg·ha^−1^).

### 2.2 Experimental details and crop husbandry practices

The study comprised 10 treatment combinations integrating three bio-stimulants, PGPR, VAM, and SWE with varying levels of the recommended dose of fertilizers (RDF). These treatments (T_1_ to T_10_) were laid out in a randomized block design with five replications. The details of the treatments along with their symbols are presented in Table 1. The selected crop varieties Mescavi (Berseem) and Avika Bajra-19 (Pearl Millet) were sown during the *Rabi* and *Summer* seasons for two subsequent years for experimentation.

**Table 1:** Experimental design and treatment details.

Treatment	Berseem-pearl millet cropping system
T_1_	Absolute control
T_2_	PGPR + VAM
T_3_	PGPR + VAM + 50% RDF
T_4_	PGPR + VAM + 75% RDF
T_5_	PGPR + VAM + 3 sprays of SWE
T_6_	PGPR + VAM + 50% RDF + 2 sprays of SWE
T_7_	PGPR + VAM + 75% RDF + 1 sprays of SWE
T_8_	100% RDF
T_9_	PGPR + VAM + 100% RDF
T_10_	PGPR + VAM + 100% RDF + 1 sprays of SWE

*RDF, Recommended dose of fertilizers; PGPR, plant growth growth-promoting rhizobacteria; VAM, vesicular arbuscular mycorrhiza; SWE, seaweed extract; SWE sprayed at 25, 35, and 45 days after sowing (DAS) for 3 sprays; 25 and 35 DAS for 2 sprays, and 25 DAS for a single spray, both in Pearl Millet-Berseem Cropping system@2 mL·L^−1^ of water.

The land was cross-ploughed with the help of a tractor-drawn disc harrow, followed by a rotavator and planking to bring the soil to a proper tilth. The field layout and bunds were made with a tractor-drawn bund former. At first, the seeds were treated with PGPR (Pusa Sampoorna)@250 mL·ha^−1^ of seed, followed by VAM fungi (Pusa Mycorrhizae)@625 g·ha^−1^ with the help of a jaggery solution. The treated seed was allowed for the shade drying for 30 min. A seed rate of 25 kg·ha^−1^ and 12 kg·ha^−1^ was used for both berseem and pearl millet, respectively. In accordance with treatments, both treated and untreated seeds were weighed separately in small pockets for each plot, and the seed was sown on the respective plots by adopting the wet bed method of sowing for berseem and manually operated seed drill for pearl millet at a spacing of 45 × 15 cm^2^.

According to the nutrient treatments, fertilizers were applied at the recommended rates of 20:60:40 kg·ha^−1^ (N:P_2_O_5_:K_2_O) for berseem and 80:30:30 kg·ha^−1^ for pearl millet. For pearl millet, one-third of the nitrogen and the full doses of phosphorus and potassium were applied at sowing (basal dose), while the remaining two-thirds of nitrogen was given in two equal splits at 25 and 40 days after sowing (DAS). The rest of the agronomic practices were adopted as per the recommendations of Indian Grassland and Fodder Research Institute, Jhansi, across all the treatments.

### 2.3 System productivity estimation

To record the green fodder yield of berseem, the crop was harvested in three sequential cuts. The first cut was taken at 60 DAS from the central rows of each plot, following the discard of a non-experimental border area. Then the second and third cuts, were taken at 40 days after the preceding cut from the same net plot area. For each cut, the harvested green fodder was weighed in kilograms per plot and subsequently converted into tonnes per hectare (t·ha^−1^). The total seasonal green fodder yield was then calculated as the sum of the yields from all three individual cuts. To determine the dry fodder yield, the green fodder yield from each cut was multiplied by its specific dry matter content, and the total dry fodder yield for berseem was derived from the summation of the dry matter yields across the cuts.

For pearl millet, the methodology for yield assessment differed. The crop was harvested at a specific growth stage, the 50% blooming phase, which occurred at 55 DAS. The harvest was conducted from a designated one-square-meter area within the net plot of each treatment. The resulting green biomass was weighed and then converted into a yield value expressed as tonnes per hectare. The dry fodder yield for pearl millet was subsequently worked out by multiplying the total green fodder yield by the crop’s dry matter content, providing a single, consolidated measure of dry matter production for the entire crop cycle by using Equation 1.


$$\mathrm{DFY}(t\cdot {\mathrm{ha}}^{-1})=\frac{\left\{\mathrm{GFY}(t\cdot {\mathrm{ha}}^{-1})\times \mathrm{Dry}\;\text{matter}(\%)\right\}}{100}$$ (1)

Then, system productivity was assessed in terms of:

(a) System total green and dry fodder yield (t·ha^−1^) was obtained by summing up the total green fodder yield and dry fodder yield of berseem and pearl millet, treatment-wise, separately.
(b) System production efficiency (kg GFY·ha^−1^·day^−1^) was calculated by dividing the system’s total green fodder yield by the crop-grown duration of berseem and pearl millet.
(c) System equivalent yield (t·ha^−1^) was calculated based on pearl millet equivalent yield.

### 2.4 Energy auditing

All inputs used during the cultivation of berseem and pearl millet and output obtained (dry fodder yield and pearl millet grain yield) were used for estimating the energy relations. Energy in agriculture, based on its release pattern, can be classified as direct and indirect energy sources. Direct sources of energy which release energy directly upon the utilization viz., human labour, diesel, electricity and irrigation water (Ghorbani et al., 2011) are considered in present study. The radiation, rain and wind are also direct sources of energy, but were not taken in present study. Indirect sources of energy do not release energy directly, but dissipate energy during different conversion processes and these include seeds, fertilizers, herbicides, pesticides and machinery (Ghorbani et al., 2011). Indirect energy in the form of plant nutrients absorbed by crops from the soil or energy involved in increased or decreased of soil organic matter was not considered in present study.

Energy based on resources is classified into two types. First is renewable energy which includes human labour, irrigation water and seeds, whereas another one is non- renewable energy that includes diesel, electricity, fertilizers, herbicides and machinery (Yilmaz et al., 2005; Ghorbani et al., 2011).

The energy coefficients used for assessment of energy outputs and inputs for every item and crop production technologies by considering their primary data are given in Table 2. All input energy equivalents were summed to get an anticipated total input energy. Energy consumption in different crop management operations was calculated on the basis of energy utilized in field preparation, sowing, fertilizer application, irrigation, herbicide application/ weeding, plant protection and harvesting.

**Table 2:** Energy equivalents of inputs and outputs used for fodder berseem-pearl millet cropping system.

Sr. no.	Particulars	Unit	Energy equivalent (MJ·unit^−1^)	References
1	Seeds	kg	14.7	Parihar et al. (2013)
2	Human labour	hr	1.96	Devasenapathy et al. (2009)
3	Farm machinery	kg	62.7	Mittal and Dhawan (1988)
4	Diesel	L	56.31	Parihar et al. (2018)
5	Electrical motor	kg	64.8	Devasenapathy et al. (2009)
6	Sickle	hr	0.836	Nassiri and Singh (2009)
7	Sprayer	hr	0.50	Nassiri and Singh (2009)
8	FYM	kg	0.3	Parihar et al. (2013)
9	N	kg	60.6	Toader and Lăzăroiu (2014)
10	P	kg	11.1	Toader and Lăzăroiu (2014)
11	K	kg	6.7	Devasenapathy et al. (2009)
12	PGPR/VAM/biofertilizer	kg	2.98	Mihov et al. (2012)
13	Seaweed extract	kg	0.70	Milledge et al. (2014)
13	Irrigation	m^3^	1.02	Lal et al. (2019)
14	Herbicide	L	288	Chaudhary et al. (2006)
15	Insecticide	L	237	Khosruzzaman et al. (2010)
16	Fungicide	L	196	Khosruzzaman et al. (2010)
Output				
17	Dry fodder (Berseem)	kg	18	Mittal et al. (1985)
18	Grain pearl millet	kg	14.7	Nassiri and Singh (2009)
19	Stover pearl millet	kg	12.5	Chaudhary et al. (2017)

The amount of energy produced from the biomass (dry fodder yield) of berseem and pearl millet (grain and stover yield) crops was also computed in terms of energy by using corresponding energy coefficients (Table 2). Energy use indices were calculated as per the procedure given by Mittal and Dhawan (1988) by using Equations 2-9.


$$\text{Energy Output}(\mathrm{MJ}\cdot {\mathrm{ha}}^{-1})=\mathrm{DFY}\;(\mathrm{kg}\cdot {\mathrm{ha}}^{-1})\times 18$$ (2)

$$\begin{array}{c} \mathrm{Net}\;\text{Energy}(\mathrm{MJ}\cdot {\mathrm{ha}}^{-1})=\text{Energy output}(\mathrm{MJ}\cdot {\mathrm{ha}}^{-1})\\ -\text{Energy input}(\mathrm{MJ}\cdot {\mathrm{ha}}^{-1}) \end{array}$$ (3)

$$\text{Energy Ratio}=\frac{\text{Energy Output}(\mathrm{MJ}\cdot {\mathrm{ha}}^{-1})}{\text{Energy Input}(\mathrm{MJ}\cdot {\mathrm{ha}}^{-1})}$$ (4)

$$\text{Energy Productivity}=\frac{\mathrm{DFY}(\mathrm{kg}\cdot {\mathrm{ha}}^{-1})}{\text{Energy Input}(\mathrm{MJ}\cdot {\mathrm{ha}}^{-1})}$$ (5)

$$\text{Energy Profitability}=\frac{\mathrm{Net}\;\text{Energy}(\mathrm{MJ}\cdot {\mathrm{ha}}^{-1})}{\text{Energy Input}(\mathrm{MJ}\cdot {\mathrm{ha}}^{-1})}$$ (6)

$$\text{Specific Energy}(\mathrm{MJ}\cdot t^{-1})=\frac{\text{Energy Input}(\mathrm{MJ}\cdot {\mathrm{ha}}^{-1})}{\mathrm{DFY}(t\cdot {\mathrm{ha}}^{-1})}$$ (7)

$$\text{Renewable energy ratio}=\frac{\text{Energy Output}(\mathrm{MJ}\cdot {\mathrm{ha}}^{-1})}{\text{Renewable energy}(\mathrm{MJ}\cdot {\mathrm{ha}}^{-1})}$$ (8)

$$\text{Energy Intensity}(\mathrm{MJ}\;\mathrm{INR})=\frac{\;\text{Energy Output}(\mathrm{MJ}\cdot {\mathrm{ha}}^{-1})}{\text{Cost of cultivation}(\mathrm{INR}\cdot {\mathrm{ha}}^{-1})}$$ (9)

### 2.5 Estimation of system profitability

Economics for different treatments of berseem and pearl millet were worked out by taking into account the cost of inputs, operations, and price of output prevailing at Bareilly (UP) during the course of investigation. System cost of cultivation was calculated by summing the cost of production of berseem and pearl millet and expressed in terms of United States dollars per hectare (US$ ha^−1^). Likewise, the gross returns were worked out by considering the prevailing market price of berseem and pearl millet at the time of harvest and yield and then system gross returns were obtained by summing GR of berseem and pearl millet by using Equation 10. The net returns were calculated by deducting the cost of cultivation from the gross returns as per treatments by using Equation 11. Benefit: Cost ratio was worked out by using Equation 12:


$$\begin{array}{c} \text{Gross Returns}(\mathrm{US}\text{\$}\cdot {\mathrm{ha}}^{-1})=\text{Yield}(t\cdot {\mathrm{ha}}^{-1})\\ \times \frac{\text{Price of green fodder}}{\text{Grain}/\text{Stover}} \end{array}$$ (10)

$$\begin{array}{c} \mathrm{Net}\;\text{Returns}(\mathrm{US}\text{\$}\cdot {\mathrm{ha}}^{-1})=\text{Gross returns}\\ -\text{Total cost of cultivation} \end{array}$$ (11)

$$B:C\;\;\text{ratio}=\frac{\text{Gross Returns}(\mathrm{US}\text{\$}\cdot {\mathrm{ha}}^{-1})}{\text{Total cost of cultivation}(\mathrm{US}\text{\$}\cdot {\mathrm{ha}}^{-1})}$$ (12)

### 2.6 Statistical analysis

The experimental data were subjected to analysis of variance (ANOVA) appropriate to the experimental design to evaluate the significance of treatment effects. When treatment effects were found to be significant at the 5% probability level (*p* ≤ 0.05), mean separation was performed using the Least Significant Difference (LSD) test. Treatment means are presented graphically as bar diagrams with standard error (±SE) bars, and different letters indicate statistically significant differences among treatments according to the LSD test at *p* ≤ 0.05.

## 3 Results and discussion

### 3.1 System productivity

#### 3.1.1 System fodder yields

System productivity of the berseem–pearl millet cropping system was significantly influenced by the application of bio-stimulants and graded levels of RDF (Table 3). Across both years, the highest green fodder yield (GFY) and dry fodder yield (DFY) were recorded under T₁₀ (PGPR + VAM + 100% RDF + one SWE spray), producing mean GFY of 80.81 t·ha^−1^ and DFY of 14.49 t·ha^−1^, which was statistically at par with T_9_ (PGPR + VAM + 100% RDF). These treatments significantly outperformed the conventional fertilization practice (T_8_: 100% RDF), registering yield advantages of about 6.9% in GFY and nearly 20% in DFY. Treatments integrating bio-stimulants with reduced fertilizer levels, particularly T_4_ and T_7_ (75% RDF + PGPR + VAM with/without SWE), produced yields comparable to T_8_, indicating the possibility of reducing chemical fertilizer inputs without compromising system productivity. The lowest yields were consistently observed under the absolute control (T_1_).

**Table 3:** Effect of seed treatment and foliar spray of bio stimulants on berseem-pearl millet system productivity.

Treatments	System GFY (t·ha^−1^)			System DFY (t·ha^−1^)			Production efficiency (kg GFY·ha^−1^·day^−1^)			System equivalent yield (qt·ha^−1^)		
	2023-24	2024-25	Mean	2023-24	2024-25	Mean	2023-24	2024-25	Mean	2023-24	2024-25	Mean
T_1_-absolute control	49.42^f^	47.57^f^	48.49^f^	5.91^f^	5.62^f^	5.77^f^	214.86^f^	206.82^f^	210.84^f^	29.24^f^	28.44^f^	28.84^f^
T_2_-PGPR + VAM	60.27^e^	60.54^e^	60.4^e^	7.89^e^	7.96^e^	7.93^e^	262.06^e^	263.2^e^	262.63^e^	39.11^e^	39.5^e^	39.3^e^
T_3_-PGPR + VAM + 50% RDF	68.95^c^	69.36^c^	69.15^c^	10.26^c^	10.37^c^	10.32^c^	299.78^c^	301.56^c^	300.67^c^	46.08^c^	46.54^c^	46.31^c^
T_4_-PGPR + VAM + 75% RDF	75.24^b^	75.66^b^	75.45^b^	11.99^b^	12.11^b^	12.05^b^	327.14^b^	328.94^b^	328.04^b^	51.85^b^	52.35^b^	52.1^b^
T_5_-PGPR + VAM + 3 sprays of SWE	63.53^d^	64.1^d^	63.82^d^	8.65^d^	8.77^d^	8.71^d^	276.21^d^	278.71^d^	277.46^d^	41.94^d^	42.56^d^	42.25^d^
T_6_-PGPR + VAM + 50% RDF + 2 sprays of SWE	69.21^c^	69.71^c^	69.46^c^	10.36^c^	10.47^c^	10.41^c^	300.92^c^	303.07^c^	302^c^	46.4^c^	46.95^c^	46.68^c^
T_7_-PGPR + VAM + 75% RDF + 1 sprays of SWE	75.44^b^	75.78^b^	75.61^b^	12.04^b^	12.17^b^	12.1^b^	327.98^b^	329.49^b^	328.74^b^	51.89^b^	52.34^b^	52.11^b^
T_8_-100% RDF	75.44^b^	75.75^b^	75.6^b^	12.03^b^	12.11^b^	12.07^b^	328.01^b^	329.34^b^	328.67^b^	52.02^b^	52.47^b^	52.25^b^
T_9_-PGPR + VAM + 100% RDF	80.43^a^	80.89^a^	80.66^a^	14.33^a^	14.53^a^	14.43^a^	349.7^a^	351.7^a^	350.7^a^	56.84^a^	57.44^a^	57.14^a^
T_10_-PGPR + VAM + 100% RDF + 1 sprays of SWE	80.56^a^	81.06^a^	80.81^a^	14.4^a^	14.59^a^	14.49^a^	350.26^a^	352.42^a^	351.34^a^	57.05^a^	57.67^a^	57.36^a^
LSD (*p* < 0.05)	1.92	1.92	1.77	0.37	0.29	0.30	8.35	8.35	7.71	1.42	1.35	1.22

*Same letter within each column indicates a non-significant difference among the treatments using the LSD test (*p* < 0.05). **RDF, Recommended dose of fertilizers; PGPR, plant growth growth-promoting rhizobacteria; VAM, vesicular arbuscular mycorrhiza; SWE, seaweed extract; SWE sprayed at 25, 35, and 45 days after sowing (DAS) for 3 sprays; 25 and 35 DAS for 2 sprays, and 25 DAS for a single spray, both in Pearl Millet-Berseem Cropping system@2 mL·L^−1^ of water.

#### 3.1.2 System production efficiency

System production efficiency varied significantly among treatments across both years (Table 3). The highest mean production efficiency was recorded under T_10_ (PGPR + VAM + 100% RDF + one spray of SWE; 351.34 kg GFY·ha^−1^·day^−1^), which remained statistically at par with T_9_ (PGPR + VAM + 100% RDF; 350.70 kg GFY·ha^−1^·day−1) and significantly superior to all other treatments. Treatments integrating bio-stimulants with 75% RDF, namely T_7_ (328.74) and T_4_ (328.04), exhibited production efficiency comparable to the recommended 100% RDF (T_8_: 328.67). Moderate efficiencies were observed under T_6_ (302.00) and T_3_ (300.67), where bio-stimulants were combined with 50% RDF. The lowest production efficiency was consistently recorded under the absolute control (T_1_: 210.84), indicating the critical role of nutrient and bio-stimulant interventions in enhancing daily biomass accumulation.

#### 3.1.3 System equivalent yield

System equivalent yield (SEY) was significantly influenced by the integrated application of PGPR, VAM), SWE, and graded levels of RDF during both years of experimentation (Table 3). The highest mean SEY was recorded under T_10_ (PGPR + VAM + 100% RDF + one SWE spray; 57.36 q·ha^−1^), closely followed by T_9_ (PGPR + VAM + 100% RDF; 57.14 q·ha^−1^), both of which were statistically superior to the sole application of 100% RDF (T_8_: 52.25 q·ha^−1^). Treatments receiving 75% RDF in combination with bio-stimulants, namely T_7_ (52.11 q·ha^−1^) and T_4_ (52.10 q·ha^−1^), produced SEY values statistically at par with T_8_, indicating scope for fertilizer reduction. The lowest SEY was consistently observed in the absolute control (T_1_: 28.84 q·ha^−1^).

### 3.2 Energy auditing under berseem-pearl millet cropping system

#### 3.2.1 Energy input consumption pattern of berseem and pearl millet cropping system

Energy auditing of the berseem–pearl millet cropping system showed a clear increase in total energy input with increasing intensity of nutrient management (Tables 4, 5). Total energy input ranged from 22,026 MJ·ha^−1^ in the absolute control (T_1_) to the highest value of 27,774 MJ·ha^−1^ in T_10_, which received PGPR, VAM, seaweed extract, and 100% RDF. Across treatments, operational inputs dominated the energy budget, with electricity (7,689 MJ·ha^−1^; 27.7–34.9%) and diesel (5,631 MJ·ha^−1^; 20.3–25.6%) accounting for the largest share, mainly due to irrigation and land preparation. Variations among treatments were primarily driven by nutrient-related energy inputs. Treatments receiving 100% RDF (T_8_, T_9_, T_10_) recorded the highest fertilizer energy input (5,650 MJ·ha^−1^), whereas integrated treatments with reduced RDF substantially lowered fertilizer-associated energy consumption. Notably, T_3_ (PGPR + VAM + 50% RDF) reduced fertilizer energy by nearly 50% while maintaining moderate total energy input (24,885 MJ·ha^−1^).

**Table 4:** Source-wise energy input consumption (MJ·ha^−1^) under berseem-pearl millet cropping system.

Treatment	Machinery	Diesel	Electricity	Labour	Seed	Fertilizers & biofertilizers	Water	Agrochemicals	Total energy input
T_1_-absolute control	766 (3.48)	5631 (25.57)	7689 (34.91)	1207 (5.48)	515 (2.34)	0 (0.00)	6120 (27.79)	98 (0.44)	22026
T_2_-PGPR + VAM	766 (3.48)	5631 (25.56)	7689 (34.90)	1207 (5.48)	515 (2.34)	5 (0.02)	6120 (27.78)	98 (0.44)	22031
T_3_-PGPR + VAM + 50% RDF	766 (3.08)	5631 (22.63)	7689 (30.90)	1239 (4.98)	515 (2.07)	2827 (11.36)	6120 (24.59)	98 (0.39)	24885
T_4_-PGPR + VAM + 75% RDF	766 (2.91)	5631 (21.41)	7689 (29.24)	1239 (4.71)	515 (1.96)	4237 (16.11)	6120 (23.27)	98 (0.37)	26295
T_5_-PGPR + VAM + 3 sprays of SWE	777 (3.49)	5631 (25.32)	7689 (34.58)	1396 (6.28)	515 (2.32)	9.00 (0.04)	6120 (27.52)	98 (0.44)	22235
T_6_-PGPR + VAM + 50% RDF + 2 sprays of SWE	773 (3.09)	5631 (22.51)	7689 (30.73)	1364 (5.45)	515 (2.06)	2830 (11.31)	6120 (24.46)	98 (0.39)	25020
T_7_-PGPR + VAM + 75% RDF + 1 sprays of SWE	770 (2.92)	5631 (21.36)	7689 (29.17)	1301 (4.93)	515 (1.95)	4239 (16.08)	6120 (23.21)	98 (0.37)	26363
T_8_-100% RDF	766 (2.77)	5631 (20.33)	7689 (27.76)	1239 (4.47)	515 (1.86)	5643 (20.37)	6120 (22.09)	98 (0.35)	27701
T_9_-PGPR + VAM + 100% RDF	766 (2.76)	5631(20.32)	7689 (27.75)	1239 (4.47)	515 (1.86)	5648 (20.39)	6120 (22.09)	98 (0.35)	27706
T_10_-PGPR + VAM + 100% RDF + 1 sprays of SWE	770 (2.77)	5631 (20.27)	7689 (27.68)	1301 (4.68)	515 (1.85)	5650 (20.34)	6120 (22.03)	98 (0.35)	27774

*Values in parentheses indicate the per cent share of total energy input. **RDF, Recommended dose of fertilizers; PGPR, plant growth growth-promoting rhizobacteria; VAM, vesicular arbuscular mycorrhiza; SWE, seaweed extract; SWE sprayed at 25, 35, and 45 days after sowing (DAS) for 3 sprays; 25 and 35 DAS for 2 sprays, and 25 DAS for a single spray, both in Pearl Millet-Berseem Cropping system@2 mL·L^−1^ of water.

**Table 5:** Operation-wise energy input consumption (MJ·ha^−1^) under berseem-pearl millet cropping system.

Treatment	Land preparation	Seeds and sowing	Nutrient management	Irrigation	Plant protection	Harvesting
T_1_-absolute control	6063	611	0	14073	259	1020
T_2_-PGPR + VAM	6063	611	5	14073	259	1020
T_3_-PGPR + VAM + 50% RDF	6063	611	2858	14073	259	1020
T_4_-PGPR + VAM + 75% RDF	6063	611	4269	14073	259	1020
T_5_-PGPR + VAM + 3 sprays of SWE	6063	611	208	14073	259	1020
T_6_-PGPR + VAM + 50% RDF + 2 sprays of SWE	6063	611	2993	14073	259	1020
T_7_-PGPR + VAM + 75% RDF + 1 sprays of SWE	6063	611	4336	14073	259	1020
T_8_-100% RDF	6063	611	5674	14073	259	1020
T_9_-PGPR + VAM + 100% RDF	6063	611	5680	14073	259	1020
T_10_-PGPR + VAM + 100% RDF + 1 sprays of SWE	6063	611	5747	14073	259	1020

*RDF, Recommended dose of fertilizers; PGPR, plant growth growth-promoting rhizobacteria; VAM, vesicular arbuscular mycorrhiza; SWE, seaweed extract; SWE sprayed at 25, 35, and 45 days after sowing (DAS) for 3 sprays; 25 and 35 DAS for 2 sprays, and 25 DAS for a single spray, both in Pearl Millet-Berseem Cropping system@2 mL·L^−1^ of water.

#### 3.2.2 System energy indices

**Figure 1:**
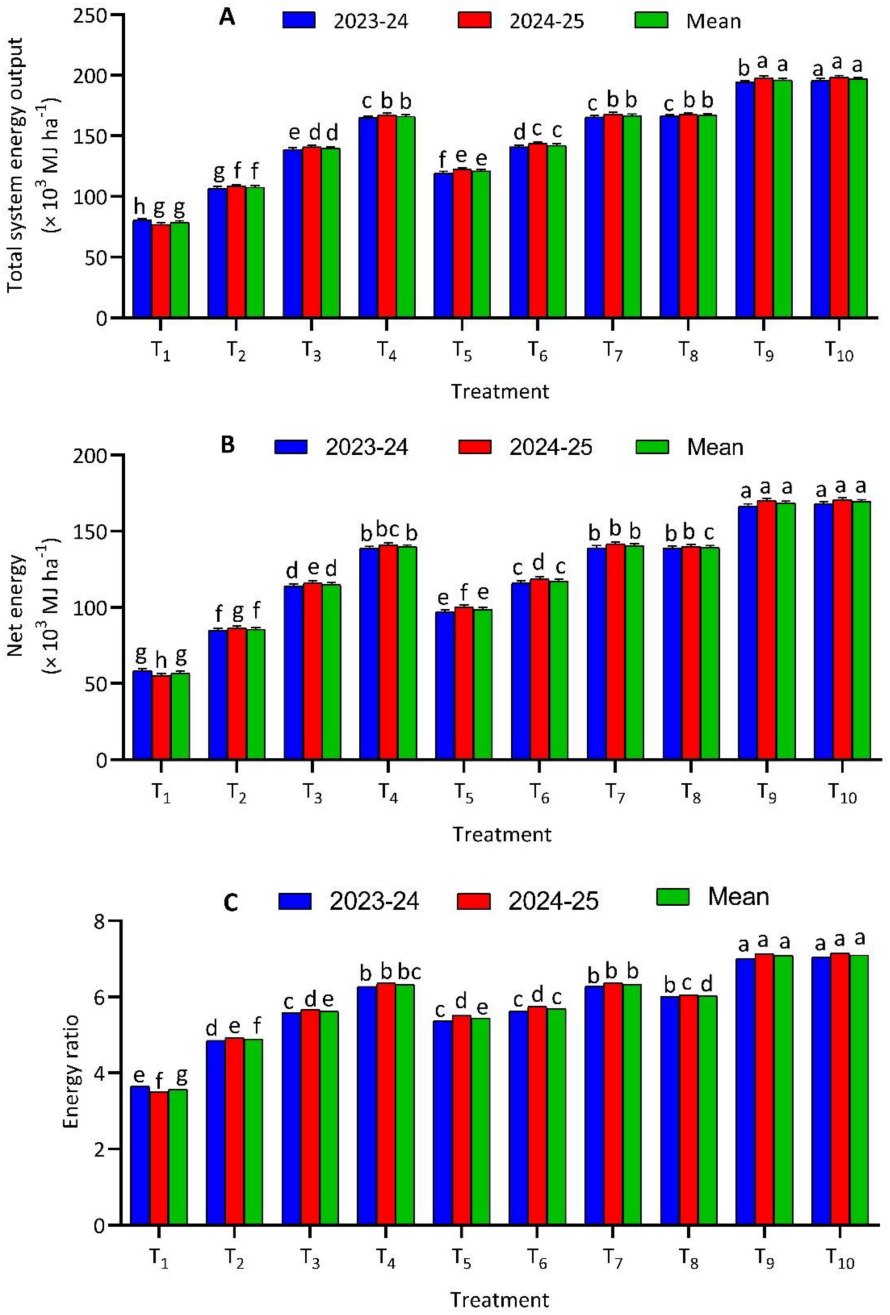
Effect of different bio-stimulant-based nutrient management strategies on total system energy output **(A)**, net energy **(B)**, and energy ratio **(C)** of berseem-pearl millet cropping system. *T_1_-Absolute control, T_2_-PGPR + VAM, T_3_-PGPR + VAM + 50% RDF, T_4_-PGPR + VAM + 75% RDF, T_5_-PGPR + VAM + 3 sprays of SWE, T_6_-PGPR + VAM + 50% RDF + 2 sprays of SWE, T_7_-PGPR + VAM + 75% RDF + 1 sprays of SWE, T_8_-100% RDF, T_9_-PGPR + VAM + 100% RDF, T_10_-PGPR + VAM + 100% RDF + 1 sprays of SWE. **RDF, recommended dose of fertilizers; PGPR, plant growth growth-promoting rhizobacteria; VAM, vesicular arbuscular mycorrhiza; SWE, seaweed extract; SWE sprayed at 25, 35, and 45 days after sowing (DAS) for 3 sprays; 25 and 35 DAS for 2 sprays, and 25 DAS for a single spray, both in Pearl Millet-Berseem Cropping system@2 mL·L^−1^ of water.

Energy indices of the berseem-pearl millet cropping system were significantly influenced by bio-stimulant–based nutrient management practices over the 2 years of study (Figure 1). The absolute control (T_1_) recorded the lowest mean total system energy output (78.75 × 10^3^ MJ·ha^−1^), net energy (56.72 × 10^3^ MJ·ha^−1^), and energy ratio (3.65), reflecting poor conversion efficiency in the absence of nutrient inputs. In contrast, treatments integrating microbial inoculants with chemical fertilizers substantially improved all energy parameters. The highest mean values were observed under T_10_ (PGPR + VAM + 100% RDF + SWE), which achieved a mean total energy output of 197.19 × 10^3^ MJ·ha^−1^, net energy of 169.41 × 10^3^ MJ·ha^−1^, and an energy ratio of 7.05, followed closely by T_9_ (PGPR + VAM + 100% RDF). Notably, T_7_ (PGPR + VAM + 75% RDF + SWE) produced energy indices statistically comparable to the sole 100% RDF treatment (T_8_), indicating efficient energy use with reduced fertilizer input.

**Figure 2:**
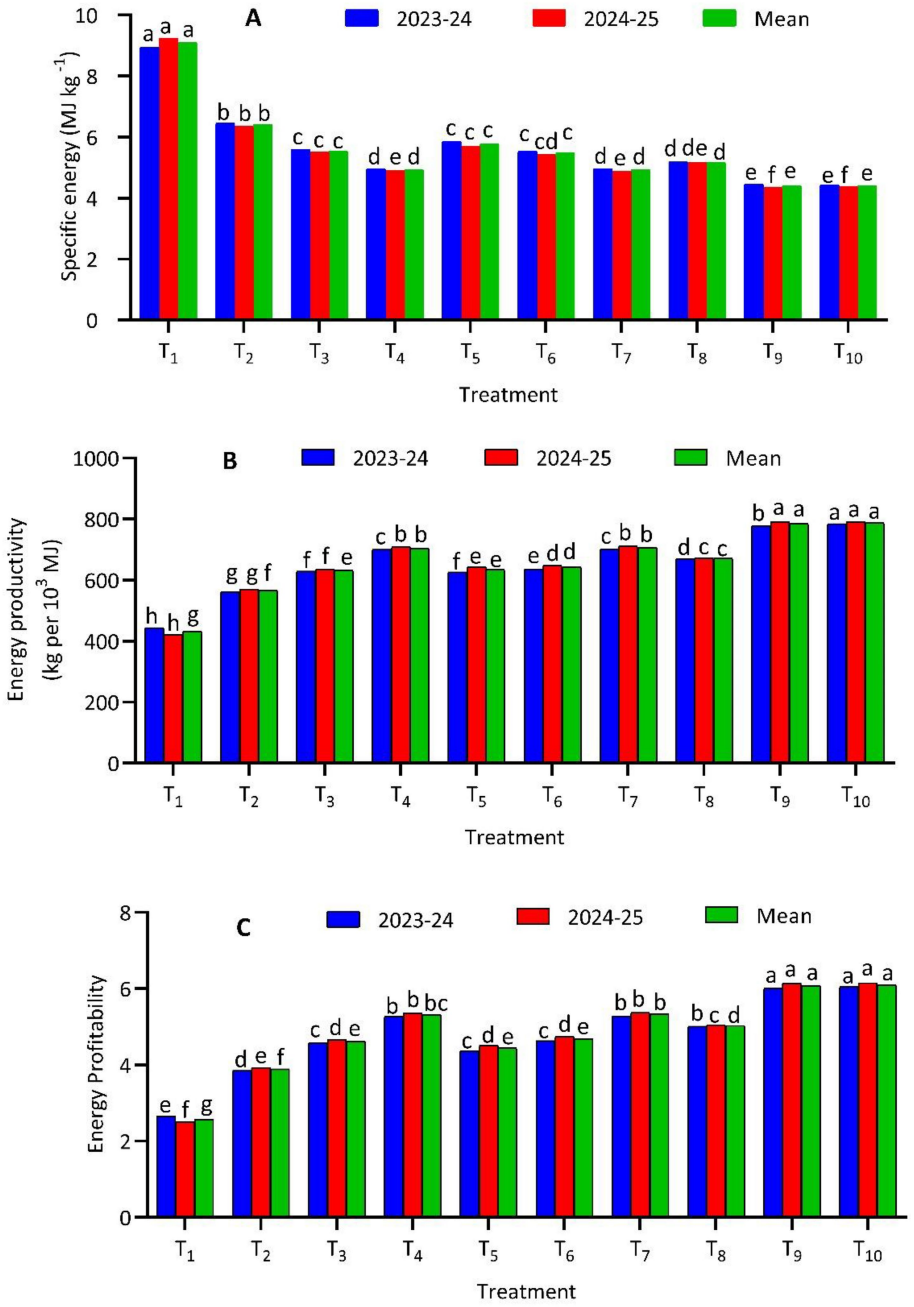
Effect of different bio-stimulant-based nutrient management strategies on specific energy **(A)**, energy productivity **(B)**, and profitability **(C)** of berseem-pearl millet cropping system. *T_1_-absolute control, T_2_-PGPR + VAM, T_3_-PGPR + VAM + 50% RDF, T_4_-PGPR + VAM + 75% RDF, T_5_-PGPR + VAM + 3 sprays of SWE, T_6_-PGPR + VAM + 50% RDF + 2 sprays of SWE, T_7_-PGPR + VAM + 75% RDF + 1 sprays of SWE, T_8_-100% RDF, T_9_-PGPR + VAM + 100% RDF, T_10_-PGPR + VAM + 100% RDF + 1 sprays of SWE. **RDF, recommended dose of fertilizers; PGPR, plant growth growth-promoting rhizobacteria; VAM, vesicular arbuscular mycorrhiza; SWE, seaweed extract; SWE sprayed at 25, 35, and 45 days after sowing (DAS) for 3 sprays; 25 and 35 DAS for 2 sprays, and 25 DAS for a single spray, both in pearl millet-berseem cropping system@2 mL·L^−1^ of water.

Energy efficiency parameters of the berseem–pearl millet cropping system were significantly influenced by bio-stimulant-based nutrient management strategies (LSD, *p* ≤ 0.05) (Figure 2). Among the treatments, T_10_ (PGPR + VAM + 100% RDF + one spray of SWE) recorded the most favorable energy indices, with the lowest mean specific energy (4.39 MJ·kg^−1^) and the highest energy productivity (787.56 kg per 10^3^ MJ) and energy profitability (6.10). This treatment was closely followed by T_9_ (PGPR + VAM + 100% RDF), indicating the strong contribution of microbial inoculants even under full fertilizer application. Both treatments significantly outperformed the conventional 100% RDF treatment (T_8_), which exhibited higher specific energy and lower productivity and profitability. The absolute control (T_1_) showed the poorest energy performance, with the highest specific energy (9.09 MJ·kg^−1^) and lowest efficiency indices. Treatments combining bio-stimulants with reduced fertilizer levels also demonstrated improved energy efficiency.

**Figure 3:**
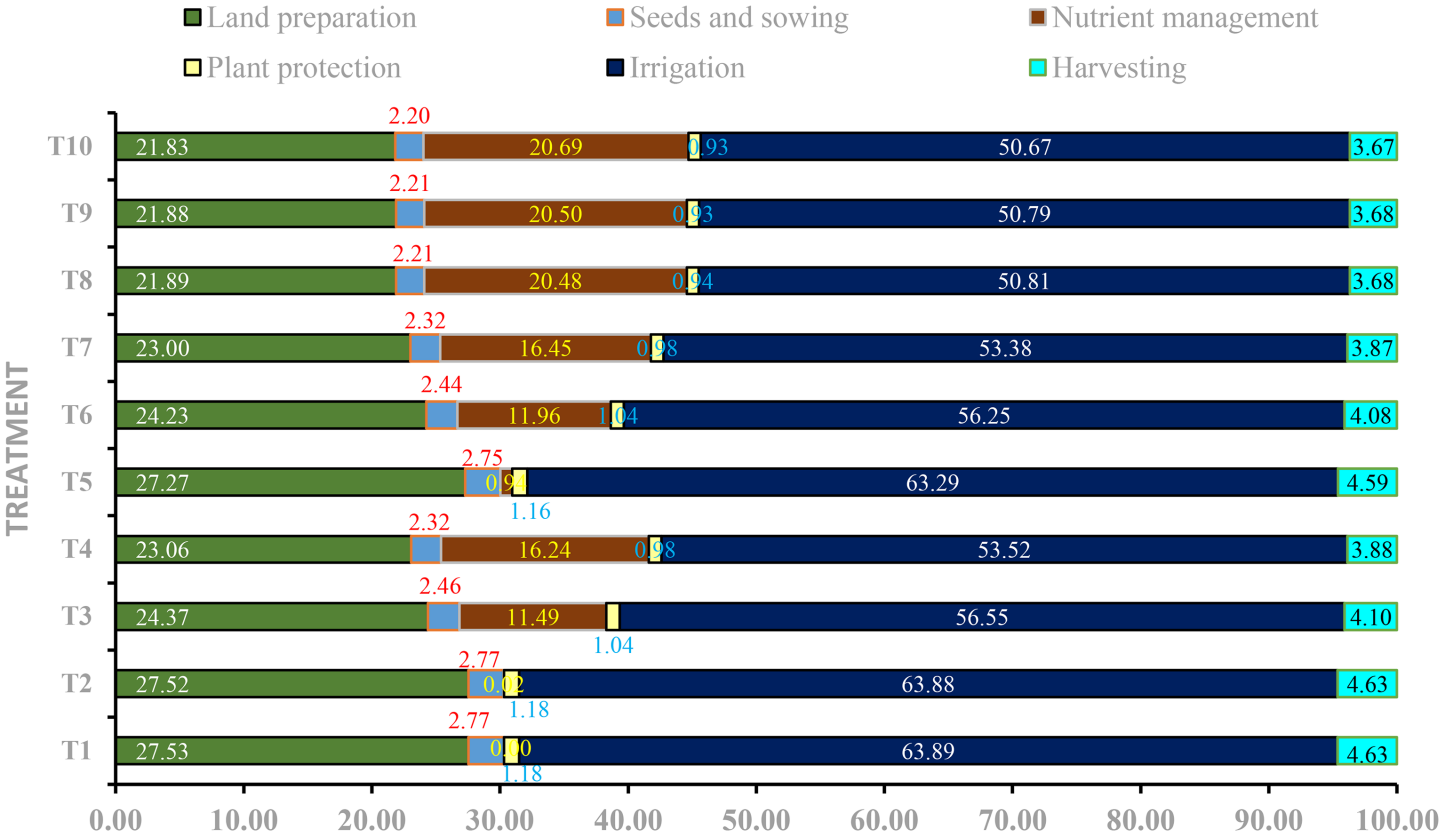
Operation-wise total energy input share (%).

Energy intensity and energy-use components varied significantly among nutrient management treatments (Figure 3). The highest energy intensity was observed in T_9_ (PGPR + VAM + 100% RDF; 6.37 MJ per 10^3^ INR), followed by T_10_ (6.17 MJ per 10^3^ INR), representing an improvement of approximately 15% over the conventional fertilization practice T_8_ (5.55 MJ per 10^3^ INR). Treatments integrating bio-stimulants with reduced fertilizer doses, namely T_4_ (5.71) and T_7_ (5.51), recorded energy intensity values statistically comparable to T_8_. Among low-input systems, the bio-stimulant-only treatment T_5_ (4.26) demonstrated a substantial improvement over the absolute control T_1_ (3.29), despite the absence of mineral fertilizers. Renewable energy ratios were highest in T_9_ (51.44) and T_10_ (51.26), whereas the maximum non-renewable energy use was also recorded under these treatments.

### 3.3 Economic profitability of berseem-pearl millet cropping system

#### 3.3.1 Cost of cultivation

Cost analysis revealed that fixed costs of cultivation remained uniform across all treatments, with a mean value of US$ 540.40 ha^−1^ over both experimental years (Table 6). In contrast, variable costs differed substantially among treatments due to variations in fertilizer, bio-stimulant, and spray inputs. The minimum mean variable cost was recorded under T_1_ (absolute control) at US$ 0.00 ha^−1^, followed by T_2_ (PGPR + VAM) at US$ 12.56 ha^−1^. Treatments receiving moderate input levels incurred intermediate variable costs, notably T_3_ (PGPR + VAM + 50% RDF; US$ 78.40 ha^−1^) and T_5_ (PGPR + VAM + three SWE sprays; US$ 89.49 ha^−1^). Higher variable costs were associated with nutrient-intensive treatments, particularly T_8_ (100% RDF; US$ 123.76 ha^−1^), T_7_ (PGPR + VAM + 75% RDF + SWE; US$ 126.82 ha^−1^), T_9_ (PGPR + VAM + 100% RDF; US$ 136.40 ha^−1^), and T_10_ (PGPR + VAM + 100% RDF + SWE; US$ 162.04 ha^−1^). Accordingly, total cultivation costs were lowest in T_1_ (US$ 540.40 ha^−1^) and T_2_ (US$ 552.97 ha^−1^) and highest in T_10_ (US$ 702.44 ha^−1^), followed by T_9_ (US$ 676.80 ha^−1^).

**Table 6:** Cost of cultivation of berseem-pearl millet cropping system as influenced by different bio-stimulants based nutrient management strategies.

Treatments	Fixed cost (USD·ha^−1^)			Variable cost (USD·ha^−1^)			Total cost (USD·ha^−1^)		
	2023-24	2024-25	Mean	2023-24	2024-25	Mean	2023-24	2024-25	Mean
T_1_-absolute control	538.50	542.30	540.40	0.00	0.00	0.00	538.50	542.30	540.40
T_2_-PGPR + VAM	538.50	542.30	540.40	12.49	12.64	12.56	550.99	554.96	552.97
T_3_-PGPR + VAM + 50% RDF	538.50	542.30	540.40	78.10	78.70	78.40	616.60	621.00	618.80
T_4_-PGPR + VAM + 75% RDF	538.50	542.30	540.40	100.58	101.80	101.19	639.08	644.10	641.59
T_5_-PGPR + VAM + 3 sprays of SWE	538.50	542.30	540.40	89.24	89.74	89.49	627.74	632.04	629.89
T_6_-PGPR + VAM + 50% RDF + 2 sprays of SWE	538.50	542.30	540.40	129.66	130.18	129.92	668.16	672.48	670.32
T_7_-PGPR + VAM + 75% RDF + 1 sprays of SWE	538.50	542.30	540.40	126.29	127.36	126.82	664.79	669.66	667.22
T_8_-100% RDF	538.50	542.30	540.40	123.28	124.23	123.76	661.78	666.53	664.16
T_9_-PGPR + VAM + 100% RDF	538.50	542.30	540.40	135.77	137.03	136.40	674.27	679.33	676.80
T_10_-PGPR + VAM + 100% RDF + 1 sprays of SWE	538.50	542.30	540.40	161.52	162.56	162.04	700.02	704.86	702.44

*Same letter within each column indicates a non-significant difference among the treatments using the LSD test (*p* < 0.05). **RDF, Recommended dose of fertilizers; PGPR, plant growth growth-promoting rhizobacteria; VAM, vesicular arbuscular mycorrhiza; SWE, seaweed extract; SWE sprayed at 25, 35, and 45 days after sowing (DAS) for 3 sprays; 25 and 35 DAS for 2 sprays, and 25 DAS for a single spray, both in Pearl Millet-Berseem Cropping system@2 mL·L^−1^ of water.

#### 3.3.2 System profitability indices

**Figure 4:**
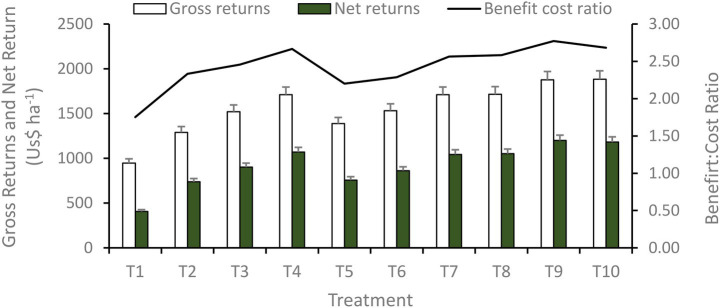
System profitability as influenced by different bio-stimulants based nutrient management strategies. *T_1_-absolute control, T_2_-PGPR + VAM, T_3_-PGPR + VAM + 50% RDF, T_4_-PGPR + VAM + 75% RDF, T_5_-PGPR + VAM + 3 sprays of SWE, T_6_-PGPR + VAM + 50% RDF + 2 sprays of SWE, T_7_-PGPR + VAM + 75% RDF + 1 sprays of SWE, T_8_-100% RDF, T_9_-PGPR + VAM + 100% RDF, T_10_-PGPR + VAM + 100% RDF + 1 sprays of SWE. **RDF, Recommended dose of fertilizers; PGPR, plant growth growth-promoting rhizobacteria; VAM, vesicular arbuscular mycorrhiza; SWE, seaweed extract; SWE sprayed at 25, 35, and 45 days after sowing (DAS) for 3 sprays; 25 and 35 DAS for 2 sprays, and 25 DAS for a single spray, both in Pearl Millet-Berseem Cropping system@2 mL·L^−1^ of water.

System-level economic analysis demonstrated that integrated use of fertilizers and bio-stimulants substantially influenced profitability parameters (Figure 4). The highest gross returns were recorded under T_10_ (US$ 1883.2 ha^−1^), reflecting its superior productivity; however, higher input costs marginally reduced net profitability. In contrast, T_9_ (PGPR + VAM + 100% RDF) achieved the maximum net returns (US$ 1199.0 ha^−1^) and benefit-cost (B:C) ratio (2.77), owing to comparable yields with relatively lower cultivation costs. Treatments receiving 75% RDF combined with bio-stimulants (T_4_ and T_7_) produced gross returns similar to sole 100% RDF (T_8_), but resulted in higher net returns and B:C ratios due to reduced fertilizer expenditure. Bio-stimulant-only treatments (T_2_ and T_5_) also generated higher net returns than the absolute control (T_1_), indicating economic benefits even under reduced input conditions.

## 4 Discussion

The superior performance of treatments integrating PGPR, VAM, RDF, and SWE highlights the synergistic role of microbial inoculants and bio-stimulants in enhancing system productivity. The highest yields under T_9_ and T_10_ can be attributed to balanced nutrient supply through 100% RDF, coupled with improved nutrient acquisition and physiological efficiency mediated by PGPR and VAM. PGPR are known to enhance biological nitrogen fixation, phytohormone synthesis, and root proliferation, thereby improving biomass accumulation (Alori et al., 2019). Concurrently, VAM fungi expand the effective root surface area via hyphal networks, improving phosphorus uptake and energy metabolism essential for fodder growth (Saleem et al., 2015; Yaseen et al., 2017). The marginal yield advantage of T_10_ over T_9_ suggests an additional stimulatory effect of SWE, likely due to the presence of bioactive compounds such as cytokinins, betaines, and micronutrients that enhance photosynthetic efficiency and stress tolerance (Craigie, 2011). Notably, treatments combining 75% RDF with bio-stimulants (T_4_ and T_7_) achieved yields statistically comparable to 100% RDF alone (T_8_), demonstrating improved nutrient-use efficiency and partial substitution of chemical fertilizers. These findings align with earlier reports indicating that integrated nutrient management enhances fodder productivity while reducing fertilizer dependency (Jat et al., 2019; Kushwah et al., 2024).

The superior system production efficiency under T_10_ and T_9_ highlights the strong synergistic interaction between PGPR, VAM, chemical fertilizers, and seaweed extract. PGPR and VAM enhance nutrient availability through biological nitrogen fixation, phosphorus solubilization, and improved root architecture, while SWE supplies natural growth regulators such as auxins and cytokinin that stimulate photosynthesis and biomass accumulation. The combined effect of these inputs improves resource-use efficiency, leading to higher daily biomass production, as also reported by (Bulgari et al., 2015). Notably, treatments T_7_ and T_4_, integrating bio-stimulants with 75% RDF, achieved production efficiencies statistically comparable to 100% RDF (T_8_). This indicates that bio-stimulants can partially substitute chemical fertilizers by improving nutrient uptake efficiency and physiological performance, thereby sustaining productivity even under reduced fertilizer inputs. In contrast, treatments with 50% RDF (T_3_ and T_6_) showed moderate gains, suggesting that nutrient supply at this level may be insufficient to fully exploit the benefits of microbial and bio stimulant activity. The consistently poor performance of the control further underscores the importance of integrated nutrient management. Similar findings on fertilizer savings through bio-stimulant integration have been documented by Kushwah et al. (2024).

The enhanced SEY under integrated nutrient management treatments highlights the synergistic role of bio-stimulants in improving crop productivity beyond conventional fertilization. The superiority of T_9_ and T_10_ over 100% RDF alone (T_8_) suggests that PGPR and VAM effectively complement mineral fertilizers by improving nutrient availability, root proliferation, and nutrient uptake efficiency. PGPR contribute through biological nitrogen fixation and phytohormone production, while VAM enhances phosphorus acquisition and water-use efficiency, collectively translating into higher system productivity (Bulgari et al., 2015). The comparable performance of 75% RDF-based treatments (T_4_ and T_7_) with 100% RDF indicates that partial substitution of chemical fertilizers with bio-stimulants can sustain yield levels while improving resource-use efficiency. The inclusion of SWE further augmented SEY, likely due to its role in stimulating physiological processes, improving stress tolerance, and enhancing nutrient assimilation. These findings corroborate earlier reports demonstrating improved yield attributes and productivity under integrated use of bio-stimulants and reduced fertilizer inputs (Li et al., 2022). Overall, the results confirm that integrated nutrient strategies can enhance SEY while offering opportunities for fertilizer savings and sustainable intensification.

The observed escalation in total energy input with increasing fertilizer application reflects the inherently energy-intensive nature of chemical fertilizer production and use. Treatments receiving 100% RDF (T_8_–T_10_) showed the highest energy inputs, primarily due to fertilizer-related energy, corroborating earlier findings that intensive input use increases energy dependence in cropping systems (Nemecek and Erzinger, 2005). However, the dominance of electricity and diesel across all treatments indicates that irrigation and tillage represent fixed structural energy costs that are largely independent of nutrient management strategies. The comparative advantage of integrated treatments lies in their ability to reduce variable energy inputs without modifying core farm operations. For instance, T_3_ (PGPR + VAM + 50% RDF) and other reduced-RDF treatments significantly lowered fertilizer energy input while maintaining comparable operational energy use. This demonstrates that bio-stimulants such as PGPR and VAM enhance nutrient-use efficiency, allowing partial substitution of chemical fertilizers and consequent energy savings. The inclusion of SWE further supports efficient nutrient assimilation, indirectly contributing to reduced reliance on energy-intensive fertilizers. Overall, integrated nutrient management treatments effectively moderated total energy input by targeting fertilizer-related energy consumption, thereby improving the energy efficiency of the system without altering its fundamental production infrastructure.

The marked improvement in energy productivity under integrated nutrient management treatments highlights the synergistic effects of bio-stimulants and inorganic fertilizers on system performance. The superior energy output and energy ratio observed in T_9_ and T_10_ compared with 100% RDF alone (T_8_) suggest that PGPR and VAM enhance nutrient-use efficiency by improving nutrient mobilization, root activity, and biological nutrient acquisition. These mechanisms reduce energy losses associated with fertilizer inputs while increasing biomass production, thereby improving net energy returns. The additional benefit of SWE in T_10_ further amplified energy efficiency, likely through enhanced physiological activity, chlorophyll synthesis, and photosynthetic efficiency, resulting in higher energy capture per unit input (Rostocki et al., 2024). Importantly, treatments combining bio-stimulants with reduced fertilizer rates demonstrated strong potential for energy-efficient intensification. T_7_, which received only 75% RDF along with PGPR, VAM, and SWE, achieved energy ratios statistically comparable to T_8_, despite lower chemical fertilizer use. This indicates that bio-stimulants can partially offset reduced fertilizer inputs without compromising system energy efficiency. In contrast, the consistently lower energy indices under the control treatment underscore the necessity of balanced nutrient inputs for sustainable energy productivity. Overall, the findings demonstrate that integrated bio-stimulant-based nutrient strategies enhance energy efficiency while enabling meaningful reductions in chemical fertilizer dependency.

The marked improvement in energy efficiency parameters under integrated bio-stimulant treatments reflects enhanced conversion of input energy into economic yield. The superior performance of T_10_ and T_9_ compared to 100% RDF alone (T_8_) suggests that the inclusion of PGPR and VAM improves nutrient use efficiency by enhancing biological nitrogen fixation, phosphorus solubilization, and root-soil interactions, thereby reducing the energy required per unit of biomass produced. The additional benefit observed in T_10_ highlights the role of seaweed extract in stimulating physiological and metabolic processes, leading to higher output per unit of energy invested (Camaille et al., 2021). The sharp decline in specific energy from the absolute control to integrated treatments demonstrates improved input-output transformation efficiency, confirming that bio-stimulants reduce dependency on energy-intensive chemical fertilizers. Importantly, treatments with reduced fertilizer input, particularly T_7_ (75% RDF + bio-stimulants), achieved energy efficiency parameters comparable to T_8_, indicating that a 25% reduction in chemical fertilizers can be compensated through biological inputs. This aligns with earlier findings that bio-stimulants enhance system efficiency and sustainability by lowering energy consumption without yield penalties (Rouphael and Colla, 2020). Overall, integrated nutrient management offers a viable pathway for improving energy profitability while reducing environmental and economic costs.

The observed increase in energy intensity under integrated nutrient management treatments reflects improved energy returns per unit economic input, primarily driven by enhanced productivity and better input-use efficiency. The superiority of T_9_ and T_10_ over the conventional 100% RDF treatment (T_8_) indicates that the inclusion of PGPR, VAM, and seaweed extract improves biological energy contributions while optimizing fertilizer use. Bio-stimulants enhance nutrient acquisition, root activity, and physiological efficiency, thereby increasing yield without proportionate increases in energy-intensive inputs. The comparable energy intensity recorded under reduced fertilizer treatments (T_4_ and T_7_) highlights the potential for lowering synthetic fertilizer inputs without compromising system energy performance. This is particularly relevant given the high embodied energy associated with nitrogen fertilizers produced via the Haber–Bosch process, along with energy expenditures related to pesticide manufacture, electricity use, and farm machinery operations (Pelletier et al., 2011; Parihar et al., 2018). Although T_9_ and T_10_ exhibited higher non-renewable energy consumption compared to T_8_, the substantially greater renewable energy ratios indicate a shift towards biologically derived and sustainable energy sources. The enhanced energy productivity and profitability observed under these treatments offset the increased non-renewable energy use. Similar improvements in energy-use efficiency under biofertilizer-integrated systems have been reported earlier by Singh et al. (2016) and Jat et al. (2023), supporting the robustness of the present findings.

The observed variation in variable and total cultivation costs across treatments primarily reflects differences in external input intensity. Uniform fixed costs indicate that baseline operational expenses were independent of nutrient management strategy, while variable costs were strongly influenced by the level of mineral fertilizers and bio-stimulant applications. Treatments relying solely on microbial inoculants (T_2_) incurred minimal additional costs, highlighting their economic attractiveness as low-input interventions. Similar observations have been reported for PGPR- and mycorrhiza-based nutrient strategies, which generally require lower monetary investment compared to chemical fertilizers (Bulgari et al., 2015). Conversely, treatments integrating higher doses of RDF, particularly T_9_ and T_10_, recorded the highest variable and total costs due to increased fertilizer use and SWE application. Although these treatments enhance productivity, their elevated input costs may reduce marginal economic gains under resource-constrained conditions. Importantly, intermediate treatments such as T_3_, T_4_, and T_7_ demonstrated moderate cultivation costs while maintaining competitive yield levels, suggesting a favorable balance between input expenditure and system performance. These findings support earlier studies indicating that partial substitution of mineral fertilizers with bio-stimulants can reduce production costs without proportionate yield penalties (Li et al., 2022). Overall, integrated nutrient management strategies offer flexibility for optimizing both productivity and cost efficiency in sustainable cropping systems.

The observed variation in profitability among treatments highlights the importance of nutrient-use efficiency rather than yield enhancement alone. Although T₁₀ recorded the highest gross returns, the additional cost associated with SWE application reduced its net profitability compared with T_9_, suggesting diminishing economic returns beyond a certain level of input intensification. The superior net returns and B:C ratio under T_9_ indicate that integrating PGPR and VAM with full RDF optimizes nutrient uptake and utilization, thereby maximizing economic efficiency. Similar outcomes were reported by Jat et al. (2023), who emphasized the role of integrated nutrient management in improving profitability. Notably, treatments with 75% RDF supplemented with bio-stimulants (T_4_ and T_7_) performed economically better than sole 100% RDF (T_8_), despite similar gross returns. This indicates that microbial inoculants effectively compensated for reduced fertilizer input by enhancing nutrient solubilization, root growth, and nutrient accessibility. These findings align with Pramanick et al. (2014), who reported a 25% reduction in fertilizer requirement with microbial supplementation. Furthermore, the positive net returns obtained under bio-stimulant-only treatments reinforce their economic viability in low-input systems. Comparable conclusions were drawn by Chen et al. (2023), highlighting the dual ecological and economic advantages of bio-stimulant-based nutrient strategies.

Although the present study demonstrated positive effects of bio-stimulant application on crop performance, certain limitations should be acknowledged. The most productive treatments were associated with full recommended fertilizer doses, which limits the ability to conclude whether bio-stimulants can effectively reduce chemical fertilizer requirements. Additionally, the experiment was conducted at a single location, which may restrict the generalizability of the findings across different agro-climatic regions and soil types. The evaluation was carried out over two cropping seasons (2023–2025), which may not be sufficient to assess long-term soil health dynamics, sustainability, or cumulative impacts of bio-stimulant application. Furthermore, the study focused primarily on agronomic responses, and detailed investigation into the physiological, biochemical, and microbial mechanisms underlying bio-stimulant action was beyond its scope. Therefore, multi-location trials conducted over longer timeframes, along with mechanistic studies, are recommended to validate and strengthen the broader applicability of these findings.

## 5 Conclusion

The present study demonstrated that plant bio-stimulants (PGPR, VAM, and seaweed extract) significantly improved productivity, bio-energy efficiency, and profitability of the berseem-pearl millet cropping system when integrated with inorganic fertilizers under the experimental conditions. Treatments combining bio-stimulants with the full recommended dose of fertilizers (T_9_: PGPR + VAM + 100% RDF and T_10_: PGPR + VAM + 100% RDF + SWE) produced the highest green and dry fodder yields, system equivalent yield, and net returns. Notably, the treatment integrating bio-stimulants with 75% RDF (T_7_) achieved yields, energy-use efficiency, and profitability statistically comparable to the full fertilizer treatments, suggesting the potential to reduce chemical fertilizer application by up to 25% without significant yield penalties during the study period. These findings indicate that bio-stimulant-based nutrient management may offer a promising approach for enhancing productivity and input-use efficiency in fodder-based cropping systems; however, further multi-location and long-term investigations are necessary to validate these results and to better assess sustainability and soil health implications under diverse agro-climatic conditions.

## Data Availability

The original contributions presented in the study are included in the article/supplementary material, further inquiries can be directed to the corresponding authors.
